# Confinement effect on the microcapillary flow and shape of red blood cells

**DOI:** 10.1063/5.0197208

**Published:** 2024-04-01

**Authors:** Mohammed Nouaman, Alexis Darras, Christian Wagner, Steffen M. Recktenwald

**Affiliations:** 1 Dynamics of Fluids, Department of Experimental Physics, Saarland University, 66123 Saarbrücken, Germany; 2 Physics and Materials Science Research Unit, University of Luxembourg, L-1511 Luxembourg, Luxembourg; 3 Micro/Bio/Nanofluidics Unit, Okinawa Institute of Science and Technology Graduate University, 1919-1 Tancha, Onna-son, Okinawa 904-0495, Japan

## Abstract

The ability to change shape is essential for the proper functioning of red blood cells (RBCs) within the microvasculature. The shape of RBCs significantly influences blood flow and has been employed in microfluidic lab-on-a-chip devices, serving as a diagnostic biomarker for specific pathologies and enabling the assessment of RBC deformability. While external flow conditions, such as the vessel size and the flow velocity, are known to impact microscale RBC flow, our comprehensive understanding of how their shape-adapting ability is influenced by channel confinement in biomedical applications remains incomplete. This study explores the impact of various rectangular and square channels, each with different confinement and aspect ratios, on the *in vitro* RBC flow behavior and characteristic shapes. We demonstrate that rectangular microchannels, with a height similar to the RBC diameter in combination with a confinement ratio exceeding 0.9, are required to generate distinctive well-defined croissant and slipper-like RBC shapes. These shapes are characterized by their equilibrium positions in the channel cross section, and we observe a strong elongation of both stable shapes in response to the shear rate across the different channels. Less confined channel configurations lead to the emergence of unstable other shape types that display rich shape dynamics. Our work establishes an experimental framework to understand the influence of channel size on the single-cell flow behavior of RBCs, providing valuable insights for the design of biomicrofluidic single-cell analysis applications.

## I. INTRODUCTION

Red blood cells (RBCs) are the main cellular constituent of blood and are vital in facilitating gas exchange between blood and tissues within the microcirculation. The properties of RBCs, such as their deformability and shape, have a profound impact on blood flow.^1^ At rest, human RBCs have a disk-like shape with a diameter of 8μm and a thickness of 2μm. Due to their high deformability, healthy RBCs can dynamically adapt their shape according to external flow conditions, such as the flow rate, vessel confinement, and the rheological properties of the surrounding fluid.^2–4^ In the microvascular network where vessel diameters are comparable to RBC size, RBCs flow in a single-file arrangement, and various RBC shapes have been observed.^5–7^ Consequently, the study of single-cell RBC flow has been conducted experimentally in microfluidic devices,^8–15^ as well as through numerical simulations,^16–21^ to understand the impact of intrinsic cell properties and external flow conditions on cell shape. Understanding single-cell RBC flow and deformation has contributed to the advancement and development of lab-on-a-chip technologies for RBC deformability analysis of storage lesions in transfusion medicine^22–24^ and for evaluation of biomedical RBC properties in health and disease.^25–27^ Nevertheless, fundamental knowledge of how external flow conditions such as channel confinement modify the RBC shape in *in vitro* microscale flows is still missing.

**FIG. 1.**
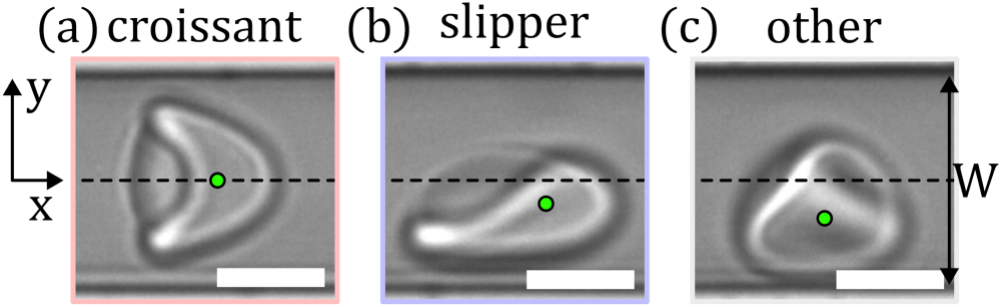
Representative RBC shapes in confined flows. (a) Centered croissant-like shape, (b) off-centered slipper-like shape, and (c) other shape in a microfluidic channel with W=10μm and H=8μm. Flow is from left to right. Black dashed lines correspond to the channel centerline across the channel width W. Green dots indicate the cell’s center of mass with respect to the y-direction. (Scale bars, 5μm.)

In microscale single-cell flow, RBCs display a wide variety of stable and dynamic shapes depending on the biophysical cell properties, channel confinement, flow velocity, and the properties of the surrounding medium. Various dynamical states, including snaking, tumbling, swinging, and tank-treading cells, have been reported under steady flow conditions.^1,28,29^ Notably, in strongly confined rectangular channels with channel dimensions (height and width) similar to the RBC size, this RBC shape complexity consolidates into two dominant RBC shapes: the croissant and the slipper shape.^12,19,24^ The symmetric croissant-like shape [Fig. 1(a)] predominantly appears at low flow velocities, while the asymmetric slipper shape [Fig. 1(b)] emerges for higher cell velocities. Guckenberger *et al*.^19^ investigated these two dominant RBC shapes in a rectangular microfluidic channel with a width of 12μm and a height of 10μm through a combination of microfluidic experiments and numerical simulations. They introduced the concept of the so-called RBC shape phase diagram, which shows the fraction of these stable croissant and slipper shapes, as well as a class called “others” [Fig. 1(c)] that were not uniquely identifiable, as a function of the cell velocity v.^19^ In their rectangular channel, they observed that the croissant shape dominated at cell velocities v<5mm/s, while roughly 70%-80% of the RBCs exhibited a slipper shape above v>5mm/s. They also reported a strong flow-shape coupling of the highly deformable RBCs and showed that the cell’s equilibrium position in the cross section and along the channel width is inherently coupled to its shape. Hence, croissant-like RBCs preferentially flow at a central position in the channel centerline, whereas slippers flow closer to the channel sidewalls (see green marker in Fig. 1). Furthermore, the authors demonstrated that the emergence of RBC shapes is influenced not only by system parameters, such as flow velocity or channel size, but also by initial conditions, including the initial shape of the RBC and its position within the channel cross section at the onset of the microfluidic channel.^19^

Recently, microfluidic investigations have unveiled the sensitivity of the stable croissant and slipper shapes in relation to biophysical cell properties such as RBC age and reduced membrane deformability.^15^ Utilizing RBCs from healthy donors and employing density gradient centrifugation methods, it has been demonstrated that the proportion of stable asymmetric, off-centered slipper-like cells diminishes with increasing age, while aged cells exhibit an augmented prevalence of stable symmetric croissants along the microchannel centerline. Consequently, analysis of the RBC shape phase diagram facilitates the discrimination of distinct RBC sub-populations, notably revealing variations in cell age.

Expanding upon the shape phase diagram proposed by Guckenberger *et al*.,^19^ additional RBC shapes in microcapillary flow, such as sphero-echinocytes, acanthocytes, and complementary pathological croissant and slipper shapes, have been introduced under pathological conditions to complement the phase diagram.^30,31^ Integrating the analysis of the cell’s equilibrium position, these cell shape classification approaches have been recently applied to assess changes in the microcapillary flow behavior of neuroacanthocytosis syndrome and COVID-19 patients and patients undergoing dialysis,^24,30–32^ demonstrating its potential as a biomarker and functional diagnostic tool for specific pathologies and to evaluate the quality of stored blood.

In addition to experiments conducted in rectangular microchannels, numerical simulations revealed a diverse range of steady and dynamic RBC shapes in round microcapillaries. Fedosov *et al*.^16^ constructed a comprehensive shape phase diagram encompassing stationery parachutes, swinging slippers, tumbling, and snaking discocytes as a function of the channel confinement and the shear rate in the system with a circular cross section. Note that parachute-shaped RBCs, which tend to become more asymmetric in rectangular channels, are often referred to as croissants in microfluidic experiments with square or rectangular microchannels. Similar to experimental observations,^19^ parachute-like RBCs in the round capillaries flow in the tube center, while slippers are displaced further from the channel center.^16^ The conditions of occurrence of these shapes and the transition between the dynamic shapes critically depend on the RBC properties and the viscosity contrast between RBC cytosol and blood plasma.^21^

While experimental and numerical progress has advanced our understanding of how intrinsic cell properties, such as their deformability, cytosol viscosity, viscoelasticity of the membrane, and age, affect the RBC shape and microscale flow behavior,^15,33,34^ detailed experimental investigations of how slight changes in the channel confinement affect the RBC capillary flow and the shape phase diagram remain scarce. Presently, poly(dimethylsiloxane) (PDMS) microchannels fabricated using soft lithography techniques serve as a common platform to investigate the microscale flow of biological systems.^35,36^ These techniques generally offer highly reproducible results down to the nanoscale, with low shrinkage during cure.^37^ However, inconsistencies in PDMS casting, curing, releasing, or bonding can introduce variations in the channel dimensions between different molds or chips during fabrication. Additionally, changes in the channel cross section may occur due to the deformation of flexible PDMS microchannels under pressure-driven flow.^38–42^ Therefore, understanding the effect of channel confinement on the different RBC shapes, employed for *in vitro* microvascular flow assessment, is paramount. This becomes particularly crucial in the context of assessing microscale flow under physiologically relevant conditions, where subtle variations in channel confinement can significantly influence the observed RBC shapes and, consequently, the accuracy of the RBC shape phase diagram.

In this study, we examine the single-cell flow of RBCs through six distinct microfluidic channels. Specifically, we explore the effect of how small differences in the order of a few micrometers affect the RBC phase diagram, the cells’ equilibrium positions, their elongation, and the occurrence of unstable cell shapes. Our study aims to conduct a comprehensive experimental investigation to explore the sensitivity of these approaches, particularly how the RBC shape phase diagrams (PDs) are influenced by the precise channel dimensions. This understanding is crucial for elucidating RBC flow dynamics in more intricate systems, such as those found in pathological conditions. For this, we use high-speed imaging of microscale RBC flow and systematically vary the channel width and height, resulting in different aspect ratios and confinement ratios of the channel cross section. Our findings highlight that the presence of stable slipper-like shapes requires a non-square rectangular cross section, while croissant-like cells also disappear gradually in square channels. Intriguingly, both stable croissant and slipper shapes disappear when the channel dimensions exceed 10μm, leading to the emergence of highly dynamical other RBC shapes. Additionally, we show how the elongation of croissants and slippers, quantified by the elongation index EI, scales with the shear rate in the microchannels, highly relevant for biomicrofluidic technologies aimed at measuring RBC deformability.^27^

## II. MATERIAL AND METHODS

### A. RBC sample preparation

Blood was collected with informed consent from three healthy voluntary donors (age 28–51 years) through needle prick and it was subsequently suspended in phosphate-buffered saline solution (Gibco PBS, Fisher Scientific, Schwerte, Germany). Following collection, samples were centrifuged for 5 min at 3000 × *g*. The sedimented RBCs were washed three times with PBS and final RBC samples were adjusted at a hematocrit concentration of 1%Ht in a PBS solution that contained 1g/l bovine serum albumin (BSA, Sigma-Aldrich, Taufkirchen, Germany). The PBS/BSA mixture is a Newtonian fluid with a shear viscosity of roughly η=1.2mPas.^13,42^

Blood withdrawal, sample preparation, and microfluidic experiments were performed according to the guidelines of the Declaration of Helsinki and approved by the ethics committee of the “Ärztekammer des Saarlandes” (permission number 51/18).

### B. Microfluidic setup

**FIG. 2.**
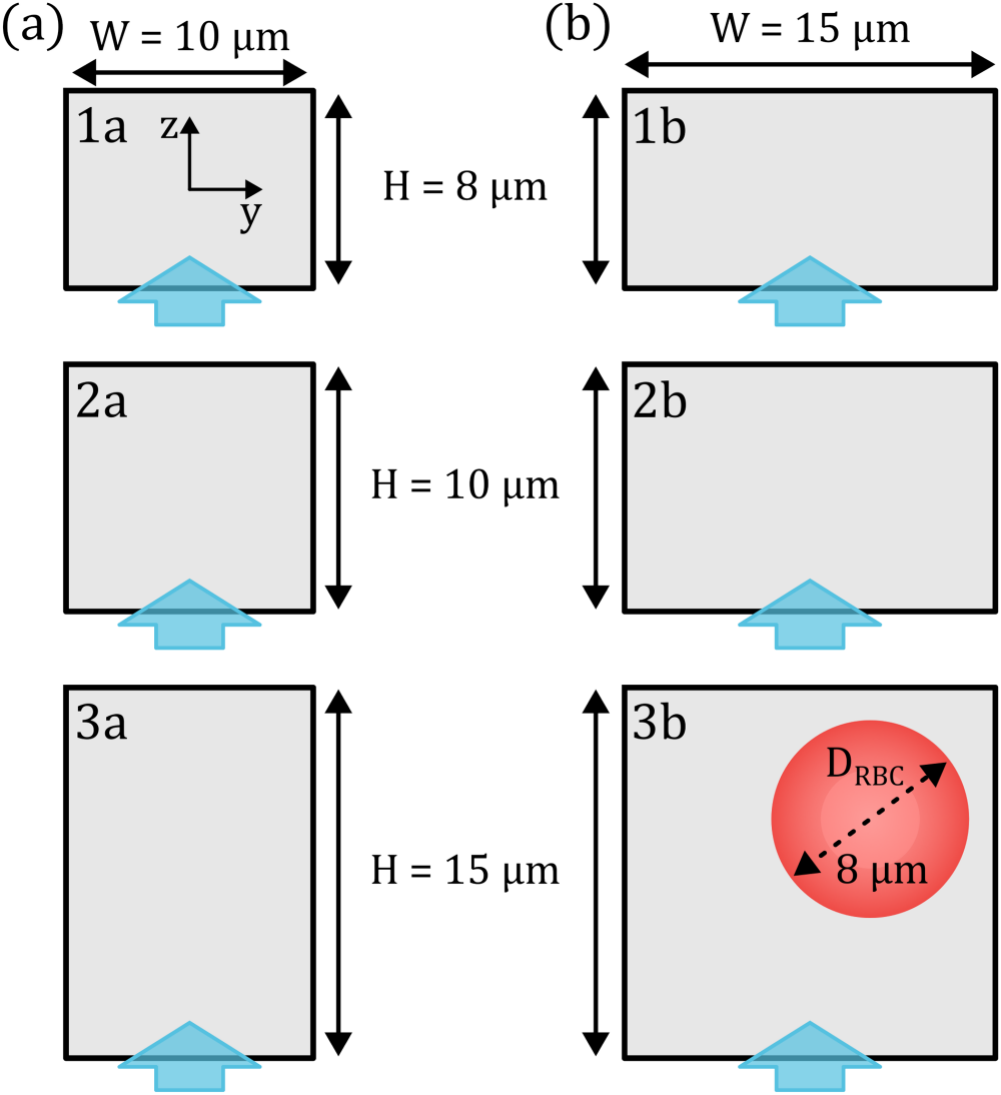
Schematic representation of the used microfluidic channels. We employ different channels with a width of (a) 10μm and (b) 15μm in combination with different channel heights. Blue arrows indicate the optical access in the microfluidic setup. The red circle illustrates the size of an undeformed discocyte-shaped RBC with a diameter of 8μm relative to the used channels.

RBC suspensions were pumped through six distinct microfluidic chips using a high-precision pressure device (OB1-MK3, Elveflow, Paris, France) to apply constant pressure drops ranging between p=50-1000mbar. The microfluidic chips were designed with microchannel having widths of either W≈10μm or W≈15μm in combination with three different heights H≈8,10,15μm (Table I and Fig. 2). The length of all microfluidic channels is L=40mm. Channel dimensions were determined by a customized MATLAB algorithm [9.14.0.2206163 (R2023a), The MathWorks, Natick, MA] that detects the channel borders based on microscopic images obtained using brightfield microscopy. Channel dimension data were averaged from different microfluidic chips as well as different positions along the channel flow direction within a chip.

**TABLE I.** Overview of the used channel geometries. All channels have a total length of L = 40 mm. Values for *W* and *H* represent mean channel dimensions and corresponding standard deviations.

Channel no.	*W*(*μm*)	*H* (*μ*m)	*AR*	*χ*
1a	10.49 ± 0.36	7.82 ± 0.29	1.40	0.89
2a	10.64 ± 0.30	10.57 ± 0.18	1.01	0.75
3a	10.39 ± 0.62	15.04 ± 0.33	0.73	0.65
1b	15.25 ± 0.32	7.78 ± 0.11	1.96	0.78
2b	15.67 ± 0.32	10.83 ± 0.18	1.45	0.62
3b	15.83 ± 0.71	15.18 ± 0.25	1.04	0.52

We define the channel aspect ratio AR=W/H and the confinement ratio $\chi =D_{RBC}/D_{h}$ with the diameter of a discocyte-shaped RBC at rest $D_{RBC}\approx 8\;\mu m$ and the hydraulic diameter of the rectangular channel $D_{h}=2WH/(W+H)$. The microfluidic chip fabrication followed standard soft lithography techniques using polydimethylsiloxane (PDMS, RTV 615A/B, Momentive Performance Materials, Waterford, NY, USA).^37^ Subsequently, the chip was bonded to a glass slide using a plasma cleaner (PDC-32G, Harrick Plasma, Ithaca, NY, USA). The inlet and the outlet of the microfluidic chips were connected with rigid medical-grade polyethylene tubing ( 0.86mm inner diameter, Scientific Commodities, Lake Havasu City, AZ, USA) to the sample and waste containers, respectively.

The microfluidic device was mounted on an inverted microscope (Eclipse TE2000-S, Nikon, Melville, NY, USA), featuring red LED illumination (SOLIS-415C, Thorlabs Inc., Newton, NJ, USA), a 60× air objective (Plan Fluor, Nikon, Melville, NY, USA) with a numerical aperture NA=1.25. RBC flow was recorded in the middle of the microchannels at L/2 using a high-speed camera (Fastec HiSpec 2G, FASTEC Imaging, San Diego, CA, USA). All microfluidic experiments were performed at $22^{{}^{\circ}}$C.

Image sequences were post-processed using a customized MATLAB algorithm. For each single RBC, we determined the center of mass of each cell in the projection plane (Fig. 1), length a in the flow direction, and diameter b along the channel width by identifying a bounding box around the RBC shape. The cell’s elongation index is calculated as EI=(a−b)/(a+b). To determine individual cell velocities, we tracked the cell position throughout the image sequence within the field of view. A frame rate of up to 400 frames per second was used to record image sequences of RBC passing the field of view. RBC shapes in flow were classified manually following the criteria established by Guckenberger *et al*.^19^ Since we did not observe inter-individual variations in the results, data were averaged between the three healthy donors. Data analysis was performed on an average of 6714 cells per donor (between 2653 and 10 635 cells).

## III. RESULTS AND DISCUSSION

### A. RBC shape phase diagrams

**FIG. 3.**
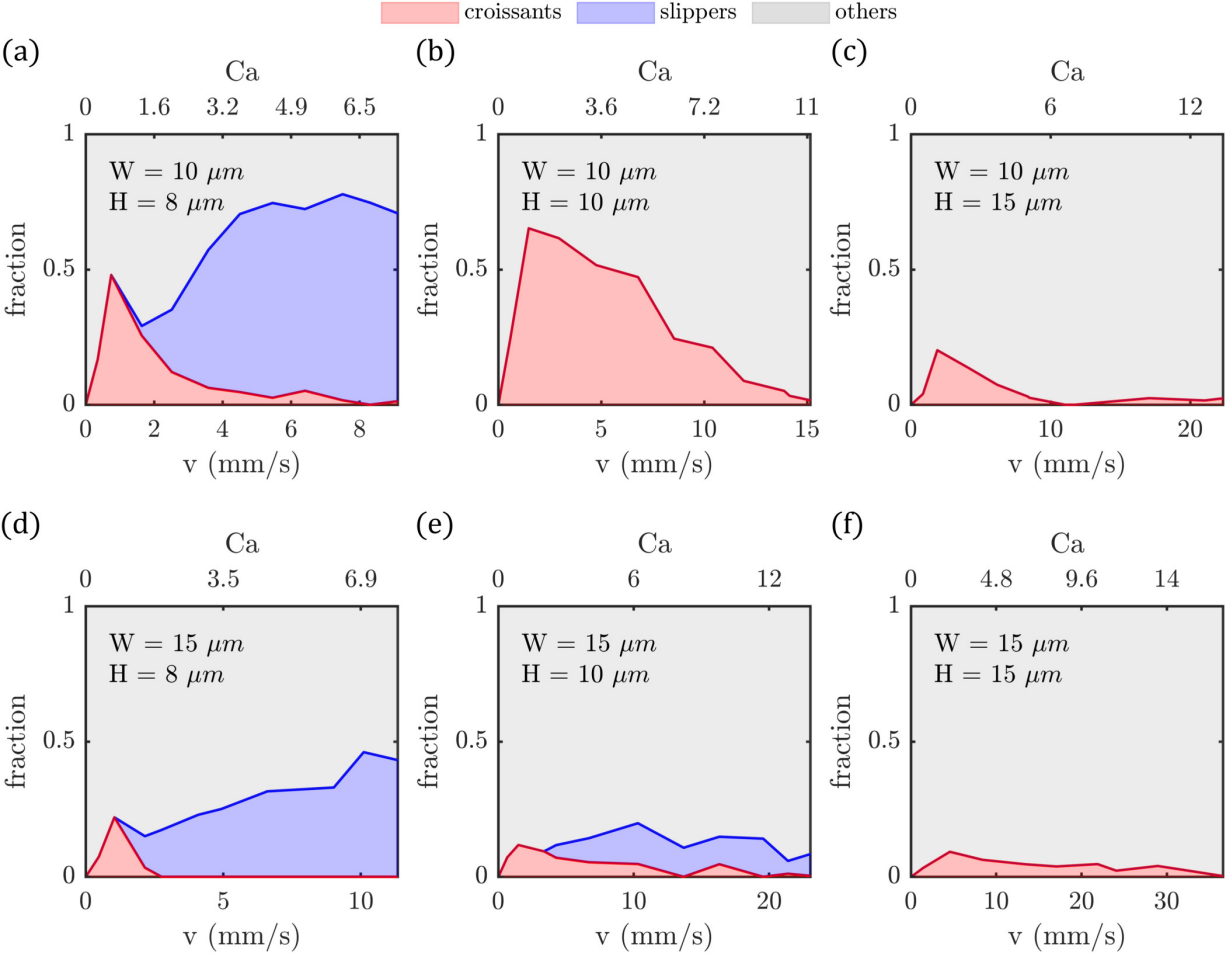
Shape phase diagrams (PDs) of RBCs in microfluidic channels: (a) W=10μm and H=8μm, (b) W=10μm and H=10μm, (c) W=10μm and H=15μm, (d) W=15μm and H=8μm, (e) W=15μm and H=10μm, and (f) W=15μm and H=15μm. Fraction of croissant-like, slipper-like, and other RBC shapes as a function of the cell velocity (bottom x axes) and capillary number Ca (top x axes) in the different microfluidic channels.

Our investigation centers on exploring the impact of channel confinement on the RBC shape in microchannels, focusing on the three dominant RBC shape classes, namely, croissants, slippers, and others, similar to the previously established phase diagram RBC shapes.^12,19,24,31^ Based on the applied pressure drop and the channel cross section, the resulting cell velocities in the microchannels are in the range of v=0.2-35mm/s (Fig. 3), with the lower range being similar to the physiological flow velocities in the microvascular network.^3,4^ Additionally, we calculated the nondimensional capillary number,

$$Ca=\frac{\eta \dot{\gamma }a}{G_{s}},$$ (1)

where η=1.2mPas is the shear viscosity of the surrounding fluid,^13,42^ $\dot{\gamma }=6v/D_{h}$ the wall shear rate in the channel,^43^ $a=D_{RBC}/2$ the discoid radius of the RBC, and $G_{s}$ the membrane shear elastic modulus.^44^ We use $G_{s}=4\;\mu N/m$, in agreement with previous studies.^45,46^ In the investigated cell velocity regime, we find Ca≈0.28-17 (see top x axes in Fig. 3).

In the RBC shape phase diagrams, we observe a prominent proportion of stable croissant and slipper-shaped RBCs for the smallest channel cross section [Fig. 3(a)] consistent with studies of RBC flow in similarly confined channels.^12,19^ The croissant fraction reaches a peak value of roughly 50% at a velocity of v≈1mm/s. As the cell velocity increases, the fraction of croissant-shaped RBC continuously decreases. Simultaneously, the amount of slipper-shaped RBCs increases above v>3mm/s, eventually reaching a plateau value at 70%-75% above 5mm/s. Keeping the channel width fixed and increasing the channel height to H=10μm results in a higher fraction of croissants while slipper-shaped RBCs disappear [Fig. 3(b)]. However, with a further increase of the channel height to H=15μm, the croissant fraction decreases again, and most RBCs display other shape types [Fig. 3(c)].

In the channels with a larger width of W=15μm and a height of H=8μm, RBC also exhibit both croissant and slipper-shaped RBCs [Fig. 3(d)] and a qualitatively similar phase diagram than for the smallest channel cross section [compare Fig. 3(a)]. However, the fraction of both stable shapes notably reduces in the wider channel. The prominent croissant peak reaches merely 20% at v≈1mm/s, while the slipper plateau saturates at 30%-40% above 5mm/s. Increasing the channel height results in a successive suppression of slipper-like cells [Fig. 3(e)]. At *W* = 15 *µ*m and *H* = 15 *µ*m, the occurrence of slipper-like RBCs is ultimately suppressed completely, whereas most cells exhibit other shapes at all investigated velocities with a few croissant-like RBC remaining [Fig. 3(f)].

Taken together, our observations emphasize that an oblong rectangular cross section coupled with a shallow channel ( H<10μm) is a prerequisite for the emergence of slippers [see Figs. 3(a) and 3(d)]. Modestly increasing the channel height by 2μm to H=10μm results in a significant reduction of slipper fraction [see Fig. 3(e)]. Consequently, stable slipper-like shapes are primarily found in strongly confined, rectangular channels with a height smaller than 10μm and a confinement ratio exceeding χ≈0.9. It is noteworthy that these slippers are categorically different than the tank-treading slipper-shaped RBCs reported in numerical simulation within round capillaries.^16,21^ In the context of round capillaries, slippers are essentially absent at χ>0.7 due to the cylindrical channel geometry and mainly appear at low confinement ratios compared to their parachute-like counterpart.

In square channels, we do not observe slipper-shaped cells [see Figs. 3(b) and 3(f)], but an increase in symmetric croissant-like RBCs when the channel dimensions do not exceed 10μm, corresponding to a confinement ratio of χ=0.75 [see Fig. 3(b)]. At χ=0.52 [see Fig. 3(f)], the fraction of croissants is drastically reduced, which is similar to observations in numerical simulations, which showed that a transition between stationary parachutes and other dynamical shapes occurs at χ≈0.5-0.55 in square microchannels.^20^

Note that our microfluidic experiments are restricted to optically accessing RBC flow through one channel side (see blue arrows in Fig. 2). As a result, cells will be classified based on their projection in the optical x- y-plane in the channel (see Fig. 2). Therefore, despite having two channels with the same dimensions swapped by $90^{{}^{\circ}}$ [Figs. 3(c) and 3(e)], we observe different shape phase diagrams. For instance, slippers detected in the channel with W=15μm and H=10μm flow at off-centered position along channel width W in y-direction in the x- y-plane. In the channel with W=10μm and H=15μm, these cells would flow at off-centered positions along the channel height H in z-direction and their projections on the x- y-observation plane do not exhibit the characteristic slipper shape. Thus, such cells would be classified as others, similar to the so-called sheared croissant class, observed in previous studies.^12^ This effect emphasizes the importance of selecting not only the appropriate channel dimensions in terms of channel height and width but also their relative aspect ratio with respect to the optical access of the channel. We did not use channels with dimensions smaller than 8μm in this study because decreasing the channel size further results in the formation of tightly squeezed, symmetric bullet-like shapes that do not transition into other asymmetric or off-centered slipper-like shapes, as demonstrated for microcapillaries with diameter 4.7-6.6μm corresponding to χ=1.7-1.2.^8,9^

### B. RBC equilibrium position across the channel width

**FIG. 4.**
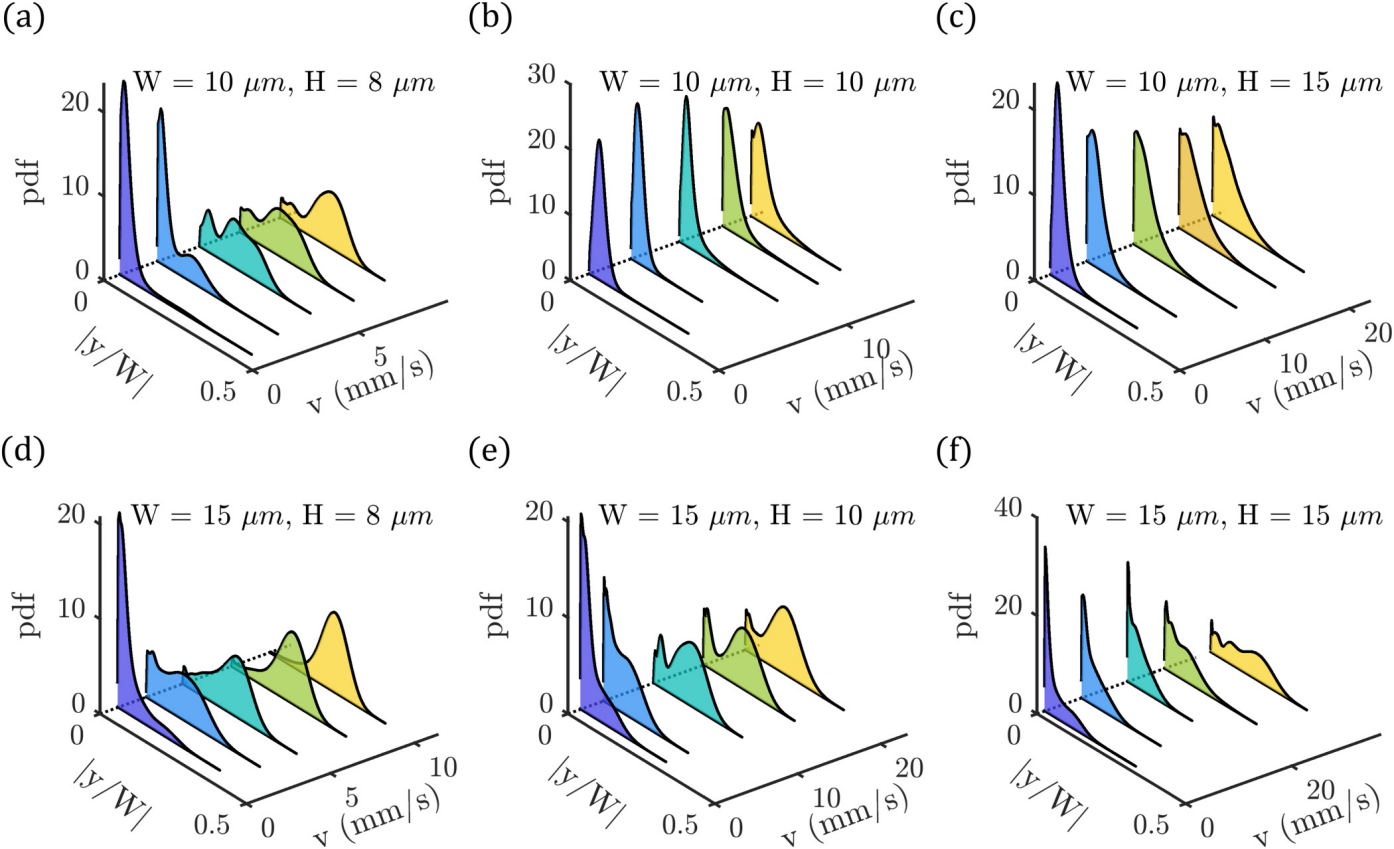
Equilibrium cell position across the channel width in the different channels: (a) W=10μm and H=8μm, (b) W=10μm and H=10μm, (c) W=10μm and H=15μm, (d) W=15μm and H=8μm, (e) W=15μm and H=10μm, and (f) W=15μm and H=15μm. Probability density distributions (pdfs) of the absolute values of the RBC y-position normalized by the channel width |y/W|. Data are shown for five velocities for the different geometries based on the data of Fig. 3.

The RBC shape is intrinsically linked to its equilibrium position in the microchannel. Based on the 2D projection of the cells in flow and the optical axes of the used setup (see blue arrows in Fig. 2), we evaluate the RBC equilibrium y-position along the channel width W. It is assessed using the probability density distributions (pdf) of the absolute value of the normalized y-coordinate |y/W| as a function of the velocity (Fig. 4).

For the smallest channel cross section with W=10μm and H=8μm [Fig. 4(a)], symmetric croissants flow at a centered position |y/W|≈0 at low velocities. Increasing the cell velocity results in a reduction of the peak at the central position and an off-centered peak emerges at |y/W|≈0.22. For the square channel [ W=H=10μm, Fig. 4(b)], most cells also flow along the channel central axis |y/W|≈0 even at high velocities exceeding 10mm/s. This pronounced centered peak in the pdfs persists when keeping the channel width fixed and increasing the height further to H=15μm, yet the pdf broadens to more off-centered cell positions at the same time [Fig. 4(c)].

In the rectangular channel with W=15μm and H=8μm [Fig. 4(d)], most cells flow at a central position |y/W|≈0 at low velocities. At velocities above 5mm/s, the pdfs exhibit only an off-centered peak at |y/W|≈0.22. Increasing the height to H=10μm [Fig. 4(e)] results in the occurrence of two pronounced peaks in the distributions at v>8mm/s, with one at the centerline and an off-centered one. Upon further increase of the channel height to 15μm [Fig. 4(f)], we still observe a central peak at low velocities up to v=20mm/s. However, RBCs do not flow at a preferred position across the channel width at higher velocities, as indicated by the broad distribution at v>25mm/s.

Our analysis of the cell’s y-position distribution aligns with the observed phase diagrams. When a significant number of slipper-shaped RBCs is observed [Figs. 3(a), 3(d), and 3(e)], a pronounced off-centered peak at |y/W|≈0.22 appears in the pdfs [Figs. 4(a), 4(d), and 4(e)]. This observation is in good agreement with previous work.^15^ Interestingly, while we find only one off-centered peak at v>5mm/s in the channel with W=15μm and H=8μm [Fig. 4(d)], two peaks at |y/W|≈0 and |y/W|≈0.22 appear for the slightly deeper channel with W=15μm and H=10μm [Fig. 4(e)]. Such pdfs with two pronounced peaks at elevated velocities have been reported before^15,31^ and are also found in the most confined channel [Fig. 4(a)] due to the emergence of both off-centered slippers and central flowing croissants and other shapes. Note that both channels [Figs. 4(a) and 4(e)] have similar channel aspect ratios AR≈1.4 (see Table I). However, the singularly peaked distribution in the channel with W=15μm and H=8μm [Fig. 4(d)] at v>5mm/s suggests that the significant fraction of other-shaped cells [Fig. 3(d)] also flow at an off-centered position, similar to the concurrently appearing slippers. These results highlight that both the RBC shape and the cell’s position in the channel cross section provide valuable information for evaluating RBC flow properties relevant to multiple clinical applications.^24^

### C. Elongation of croissants and slippers in the microchannels

**FIG. 5.**
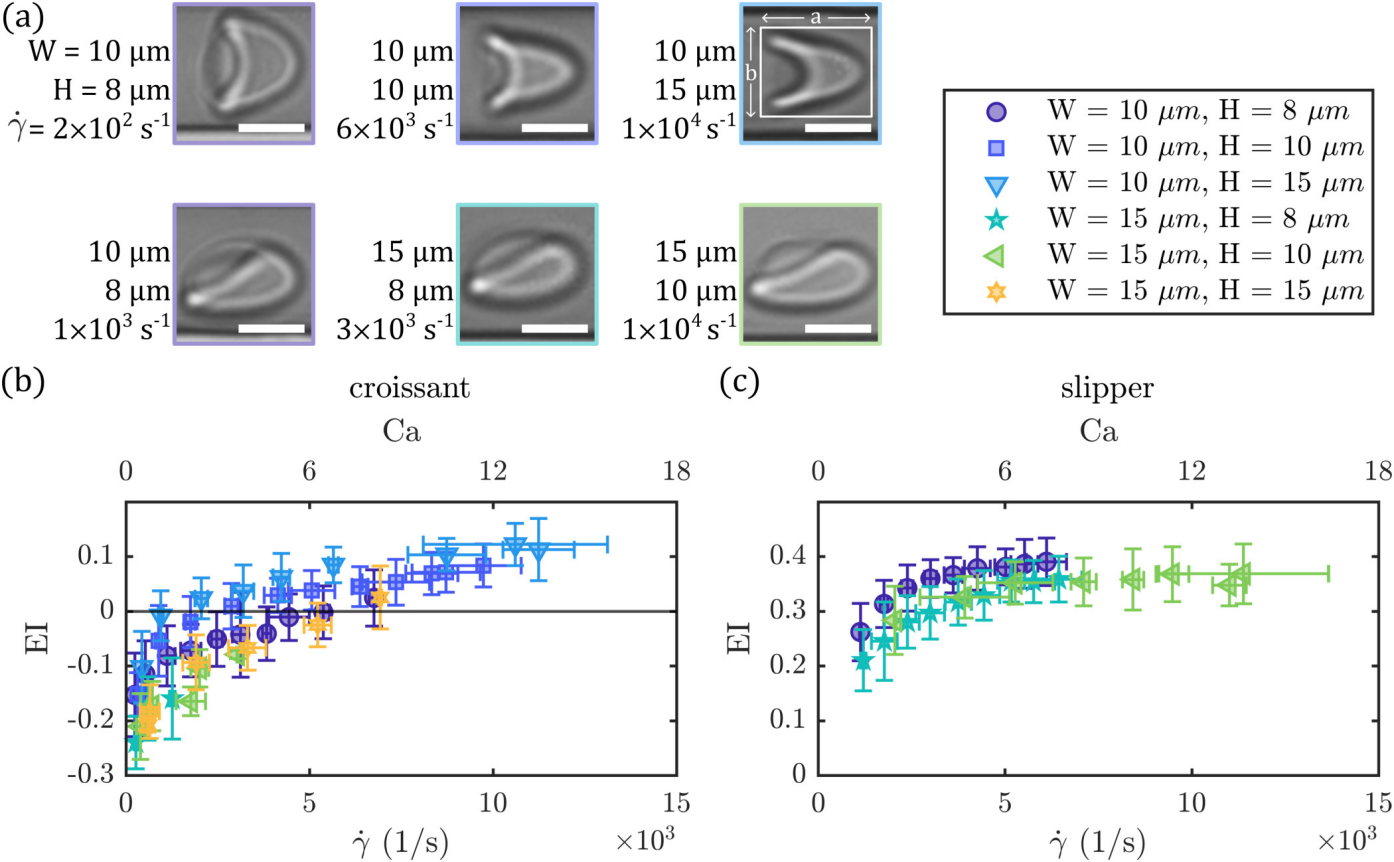
Elongation of stable croissant-like and slipper-like RBC shapes in the different channels. (a) Representative images of croissants and slippers in different channels and at various shear rates $\dot{\gamma }$. (Scale bars, 5μm.) (b) and (c) Elongation index EI=(a−b)/(a+b) in the different microfluidic channels for croissant-like and slipper-like RBCs, respectively. Data are shown as a function of the shear rate (bottom x axes) and capillary number Ca (top x axes). The length a of the RBC in the flow direction and the diameter b along the channel width are identified with a bounding box around the RBC shape, schematically shown in the top right image in (a). Error bars correspond to averaging EI data of cells at the same applied pressure drop.

In micro-confined conditions, the RBC shape critically depends on the flow rate in the channel.^11,47^ Here, we study how the shape of stable croissant and slipper-like RBCs is affected by the flow conditions in the different microchannels [Fig. 5(a)]. To characterize this effect, we assess the cell’s elongation index EI as a function of the shear rate in the channel.

At low shear rates, croissant-shaped RBCs exhibit a broad shape with a shorter length in flow direction than across the channel width, hence, EI<0 [Fig. 5(b)]. As the shear rate increases up to $\dot{\gamma }\approx 5\times 10^{3}\;s^{-1}$, there is a substantial rise of the elongation index, independent of the channel cross section. Concurrently, the increase in cell length in the flow direction leads to a transition to positive elongation indices at $\dot{\gamma }\approx 5\times 10^{3}\;s^{-1}$. Beyond $8\times 10^{3}\;s^{-1}$, cell elongation seems to saturate, eventually reaching a plateau at EI≈0.1.

For slipper-like RBCs, we also observed a gradual increase in the elongation index with the channel shear rate [Fig. 5(b)]. However, due to the initially elongated slipper shape, EI is always positive. Between $1\times 10^{3}$ and $3\times 10^{3}\;s^{-1}$, slipper-like cells exhibit an increase in EI, followed by a saturation at EI≈0.3-0.4 beyond $5\times 10^{3}\;s^{-1}$.

Assessing the elongation and stretching of RBCs by the fluid shear stress in microcapillaries has emerged as an important technology measuring RBC deformability.^25,27^ Previous work revealed differences in the dynamic deformation behavior between control RBCs and artificially stiffened RBCs, as well as RBCs of diabetes patients in microcapillary flow.^15,48,49^ In our study, the observed increase of cell elongation for stable croissants is in good agreement with previous investigations on single RBCs.^11,50^ This is attributed to the two “tails” of the croissant-shaped RBC that seem to become longer and pointed [see the top row in Fig. 5(a)] as the velocity and shear stress increase. While the cell elongation of croissants does not seem to saturate completely even at high shear rates [Fig. 5(b)], EI of slipper-shaped RBCs clearly plateaus above $\dot{\gamma }\approx 5\times 10^{3}\;s^{-1}$, similar to the previously reported high-velocity limit of cell elongation in confined microcapillaries.^9^ Future work will be required to evaluate how the observed dynamic deformation behavior of both stable RBC configurations is connected with the intrinsic cell properties, such as cytosol viscosity and membrane elasticity.

### D. Unstable other RBC shapes

In our experimental investigations, we primarily focus on stable RBC shapes in microcapillary flow. In an effort to explain the suppression of these stable shapes and the occurrence of other shapes (see Fig. 3), we assess temporal shape changes of the RBC shape within the region of interest with a length of roughly 300μm in the middle of the microfluidic chip under steady flow conditions. We consider an RBC to have an unstable shape when it rotates, tumbles, or shows any other dynamic shape transitions that lead to fluctuations $\Delta y=y(t)-\bar{y}$ of the cell’s temporal y-position y(t) from its mean y-position $\bar{y}$ larger than 5% during passage within the region of interest.

**FIG. 6.**
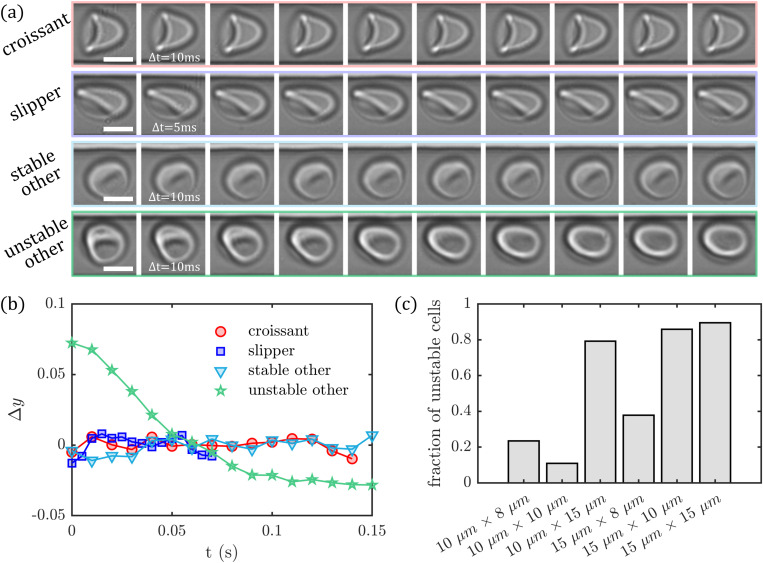
Stable and unstable other RBC shapes. (a) Representative image sequences of a croissant, a slipper, a stable other, and an unstable other cell shape in a channel with W=10μm and H=8μm. (Scale bars, 5μm.) Time intervals between consecutive images are displayed in the second image of each sequence. (b) Deviations $\Delta y=y(t)-\bar{y}$ in the lateral movement over time of the cell’s center of mass position of the four representative cells shown in (a). (c) Fraction of unstable other cells with respect to the total number of other shapes for the different channels used in this study.

Croissant and slipper-shaped RBCs exhibit stable shape configurations that do not change significantly while passing the microfluidic channel [two top rows in Fig. 6(a)]. For the other cell shape, we find a stable category [third row in Fig. 6(a)], as well as an unstable category that exhibits rich temporal dynamics [bottom row in Fig. 6(a)]. While the cell position along the channel width of the three stable shape classes does not change significantly during flow, we observe strong fluctuations of the cell’s center of mass position for the category of unstable other cell shapes [representatively shown in Fig. 6(b)]. The fraction of unstable other shapes is larger in the microfluidic channels with a large cross section, i.e., 10×15, 15×10, and $15\times 15\;\mu m^{2}$ [Fig. 6(c)].

Although our experimental field of view only covers approximately 300μm along the channel flow direction, recent cell-tracking measurements demonstrated that the croissant and slipper shapes are indeed stable.^13^ Once the cell achieves its shape after entering the microfluidic channel, it does not change under steady flow conditions. In our study, we examine the single RBC flow in the middle of the microfluidic chip at L/2=20mm. At this position, cells already reached their equilibrium y-position and final shape, and transient effects induced by the fluid inlet can be neglected.^51^ Note that the length over which tank-treading slipper-shaped RBCs in such confined channels oscillate and periodically change their y-position is usually much longer than the region of interest used in this study.^13^ Hence, tank-treading slippers are classified as a stable RBC configuration.

In contrast, the dynamic behavior of unsteady other cell shapes does not allow the RBC to reach a steady y-position [see representatively Fig. 6(b)]. These cells predominantly appear in less confined channels with χ≤0.65 [see Fig. 6(c); 10×15, 15×10, and $15\times 15\;\mu m^{2}$], which is in good agreement with numerical simulations that predict a transition from steady to dynamic shapes upon increasing the channel dimensions.^16,20,21^ In such large channels, RBCs can flow at off-centered streamlines and tumble and rotate more easily driven by the parabolic velocity profile than in the strongly confined channels. While we only classify other shapes as stable and unstable, previous work has revealed a plethora of dynamic RBC states, including tumbling, rolling, swinging, snaking, and tank-treading motions.^28,29,52^ Such dynamic RBC morphologies have received much attention as they can affect blood shear thinning behavior under microcirculatory flow conditions.^1,53^ Since approximately 20% of others exhibit unstable shapes in the channels that generate the highest fraction of stable croissants and slippers, a future research objective will be to investigate the exact nature of these shapes. For example, if tumbling trilobes or multilobes can be reliably detected within the field of measurements, this class could be integrated into the shape phase diagram. Under pathological conditions when RBC deformability or membrane mechanical properties are impaired, and various unstable states emerge, such refined shape phase diagrams represent a central role in evaluating RBC flow behavior and could be used in diagnostic applications.

## IV. CONCLUSIONS

In this study, we perform an experimental characterization of microscale RBC flow behavior, elucidating consistent RBC shape state diagrams and illustrating the complexity of RBC shapes across various confined microchannels. Based on the assessment of RBC shapes in microcapillary flow, our findings emphasize the significance of strongly confined channels, specifically with a height below 10μm and χ≈0.9 along with a rectangular cross section, to generate the characteristic croissant peak and slipper plateau at low and high velocities, respectively. Despite the potential advantages of increased throughput associated with larger channel dimensions, our results show that the number of unsteady other cell shapes that are not uniquely identifiable increases when enlarging the channel dimensions. The implications of our study are pivotal for future microfluidic designs employing RBC shape classification approaches for diagnostic purposes for specific pathologies or as quality assessments of stored blood units. In combination with recent studies highlighting the influence of intrinsic cell properties on microscale blood flow, our work underscores the critical role of confinement effects and shear rate as external conditions impacting the RBC shape, advancing our understanding of *in vitro* single RBC flow.

## Data Availability

The data that support the findings of this study are available from the corresponding author upon reasonable request.

## References

[1] L. Lanotte, J. Mauer, S. Mendez, D. A. Fedosov, J.-M. Fromental, V. Claveria, F. Nicoud, G. Gompper, and M. Abkarian, “Red cells’ dynamic morphologies govern blood shear thinning under microcirculatory flow conditions,” Proc. Natl. Acad. Sci. U.S.A. 113, 13289–13294 (2016). 10.1073/pnas.1608074113 27834220 PMC5127344

[2] Y. Suzuki, N. Tateishi, M. Soutani, and N. Maeda, “Deformation of erythrocytes in microvessels and glass capillaries: Effects of erythrocyte deformability,” Microcirculation 3, 49–57 (1996). 10.3109/10739689609146782 8846271

[3] A. R. Pries and T. W. Secomb, “Blood flow in microvascular networks,” in *Microcirculation* (Elsevier, 2008), pp. 3–36. 10.1016/B978-0-12-374530-9.00001-2

[4] T. W. Secomb, “Blood flow in the microcirculation,” Annu. Rev. Fluid Mech. 49, 443–461 (2017). 10.1146/annurev-fluid-010816-060302

[5] R. Skalak and P. I. Brånemark, “Deformation of red blood cells in capillaries,” Science 164, 717–719 (1969). 10.1126/science.164.3880.717 5778020

[6] U. Bagge, P. Brånemark, R. Karlsson, and R. Skalak, “Three-dimensional observations of red blood cell deformation in capillaries,” Blood Cells 6, 231–239 (1980). 7378593

[7] P. Gaehtgens, C. Dührssen, and K. Albrecht, “Motion, deformation, and interaction of blood cells and plasma during flow through narrow capillary tubes,” Blood Cells 6, 799–817 (1980). 7470632

[8] S. Guido and G. Tomaiuolo, “Microconfined flow behavior of red blood cells in vitro,” Compt. Rend. Phys. 10, 751–763 (2009). 10.1016/j.crhy.2009.10.002 26071649

[9] G. Tomaiuolo, M. Simeone, V. Martinelli, B. Rotoli, and S. Guido, “Red blood cell deformation in microconfined flow,” Soft Matter 5, 3736 (2009). 10.1039/b904584h

[10] J. C. A. Cluitmans, V. Chokkalingam, A. M. Janssen, R. Brock, W. T. S. Huck, and G. J. C. G. M. Bosman, “Alterations in red blood cell deformability during storage: A microfluidic approach,” Biomed Res. Int. 2014, 1–9 (2014). 10.1155/2014/764268 PMC4176636 25295273

[11] G. Tomaiuolo, L. Lanotte, R. D’Apolito, A. Cassinese, and S. Guido, “Microconfined flow behavior of red blood cells,” Med. Eng. Phys. 38, 11–16 (2016). 10.1016/j.medengphy.2015.05.007 26071649

[12] A. Kihm, L. Kaestner, C. Wagner, and S. Quint, “Classification of red blood cell shapes in flow using outlier tolerant machine learning,” PLOS Comput. Biol. 14, e1006278 (2018). 10.1371/journal.pcbi.1006278 29906283 PMC6021115

[13] S. M. Recktenwald, K. Graessel, F. M. Maurer, T. John, S. Gekle, and C. Wagner, “Red blood cell shape transitions and dynamics in time-dependent capillary flows,” Biophys. J. 121, 23–36 (2022). 10.1016/j.bpj.2021.12.009 34896369 PMC8758421

[14] A. Saadat, D. A. Huyke, D. I. Oyarzun, P. V. Escobar, I. H. Øvreeide, E. S. G. Shaqfeh, and J. G. Santiago, “A system for the high-throughput measurement of the shear modulus distribution of human red blood cells,” Lab Chip 20, 2927–2936 (2020). 10.1039/D0LC00283F 32648561

[15] M. Nouaman, A. Darras, T. John, G. Simionato, M. A. E. Rab, R. van Wijk, M. W. Laschke, L. Kaestner, C. Wagner, and S. M. Recktenwald, “Effect of cell age and membrane rigidity on red blood cell shape in capillary flow,” Cells 12, 1529 (2023). 10.3390/cells12111529 37296651 PMC10252257

[16] D. A. Fedosov, M. Peltomäki, and G. Gompper, “Deformation and dynamics of red blood cells in flow through cylindrical microchannels,” Soft Matter 10, 4258–4267 (2014). 10.1039/C4SM00248B 24752231

[17] G. R. Lázaro, A. Hernández-Machado, and I. Pagonabarraga, “Rheology of red blood cells under flow in highly confined microchannels. II. Effect of focusing and confinement,” Soft Matter 10, 7207 (2014). 10.1039/C4SM01382D 25068313

[18] D. A. Fedosov, H. Noguchi, and G. Gompper, “Multiscale modeling of blood flow: From single cells to blood rheology,” Biomech. Model. Mechanobiol. 13, 239–258 (2014). 10.1007/s10237-013-0497-9 23670555

[19] A. Guckenberger, A. Kihm, T. John, C. Wagner, and S. Gekle, “Numerical–experimental observation of shape bistability of red blood cells flowing in a microchannel,” Soft Matter 14, 2032–2043 (2018). 10.1039/C7SM02272G 29473072

[20] F. Reichel, J. Mauer, A. A. Nawaz, G. Gompper, J. Guck, and D. A. Fedosov, “High-throughput microfluidic characterization of erythrocyte shapes and mechanical variability,” Biophys. J. 117, 14–24 (2019). 10.1016/j.bpj.2019.05.022 31235179 PMC6626834

[21] A. K. Dasanna, J. Mauer, G. Gompper, A. Dmitry, and D. A. Fedosov, “Importance of viscosity contrast for the motion of erythrocytes in microcapillaries,” Front. Phys. 9, 1–13 (2021). 10.3389/fphy.2021.666913

[22] M.-E. Myrand-Lapierre, X. Deng, R. R. Ang, K. Matthews, A. T. Santoso, and H. Ma, “Multiplexed fluidic plunger mechanism for the measurement of red blood cell deformability,” Lab Chip 15, 159–167 (2015). 10.1039/C4LC01100G 25325848

[23] K. Matthews, M.-E. Myrand-Lapierre, R. R. Ang, S. P. Duffy, M. D. Scott, and H. Ma, “Microfluidic deformability analysis of the red cell storage lesion,” J. Biomech. 48, 4065–4072 (2015). 10.1016/j.jbiomech.2015.10.002 26477408

[24] S. M. Recktenwald, M. G. M. Lopes, S. Peter, S. Hof, G. Simionato, K. Peikert, A. Hermann, A. Danek, K. van Bentum, H. Eichler, C. Wagner, S. Quint, and L. Kaestner, “Erysense, a lab-on-a-chip-based point-of-care device to evaluate red blood cell flow properties with multiple clinical applications,” Front. Physiol. 13, 1–10 (2022). 10.3389/fphys.2022.884690 PMC9091344 35574449

[25] G. Tomaiuolo, “Biomechanical properties of red blood cells in health and disease towards microfluidics,” Biomicrofluidics 8, 051501 (2014). 10.1063/1.4895755 25332724 PMC4189537

[26] Y. Man, E. Kucukal, R. An, Q. D. Watson, J. Bosch, P. A. Zimmerman, J. A. Little, and U. A. Gurkan, “Microfluidic assessment of red blood cell mediated microvascular occlusion,” Lab Chip 20, 2086–2099 (2020). 10.1039/D0LC00112K 32427268 PMC7473457

[27] K. Matthews, E. S. Lamoureux, M.-E. Myrand-Lapierre, S. P. Duffy, and H. Ma, “Technologies for measuring red blood cell deformability,” Lab Chip 22, 1254–1274 (2022). 10.1039/D1LC01058A 35266475

[28] M. Abkarian, M. Faivre, and A. Viallat, “Swinging of red blood cells under shear flow,” Phys. Rev. Lett. 98, 2–5 (2007). 10.1103/PhysRevLett.98.188302 17501614

[29] J. Dupire, M. Socol, and A. Viallat, “Full dynamics of a red blood cell in shear flow,” Proc. Natl. Acad. Sci. U.S.A. 109, 20808–20813 (2012). 10.1073/pnas.1210236109 23213229 PMC3529085

[30] A. Rabe, A. Kihm, A. Darras, K. Peikert, G. Simionato, A. K. Dasanna, H. Glaß, J. Geisel, S. Quint, A. Danek, C. Wagner, D. A. Fedosov, A. Hermann, and L. Kaestner, “The erythrocyte sedimentation rate and its relation to cell shape and rigidity of red blood cells from chorea-acanthocytosis patients in an off-label treatment with dasatinib,” Biomolecules 11, 727 (2021). 10.3390/biom11050727 34066168 PMC8151862

[31] S. M. Recktenwald, G. Simionato, M. G. Lopes, F. Gamboni, M. Dzieciatkowska, P. Meybohm, K. Zacharowski, A. von Knethen, C. Wagner, L. Kaestner, A. D’Alessandro, and S. Quint, “Cross-talk between red blood cells and plasma influences blood flow and omics phenotypes in severe COVID-19,” eLife 11, 1–17 (2022). 10.7554/eLife.81316 PMC9767455 36537079

[32] F. Reichel, M. Kräter, K. Peikert, H. Glaß, P. Rosendahl, M. Herbig, A. Rivera Prieto, A. Kihm, G. Bosman, L. Kaestner, A. Hermann, and J. Guck, “Changes in blood cell deformability in chorea-acanthocytosis and effects of treatment with dasatinib or lithium,” Front. Physiol. 13, 1–11 (2022). 10.3389/fphys.2022.852946 PMC9013823 35444561

[33] F. Guglietta, M. Behr, L. Biferale, G. Falcucci, and M. Sbragaglia, “On the effects of membrane viscosity on transient red blood cell dynamics,” Soft Matter 16, 6191–6205 (2020). 10.1039/D0SM00587H 32567630

[34] A. Gürbüz, O. S. Pak, M. Taylor, M. V. Sivaselvan, and F. Sachs, “Effects of membrane viscoelasticity on the red blood cell dynamics in a microcapillary,” Biophys. J. 122, 1–12 (2023). 10.1016/j.bpj.2023.01.010 36639868 PMC10257124

[35] K. Ren, J. Zhou, and H. Wu, “Materials for microfluidic chip fabrication,” Acc. Chem. Res. 46, 2396–2406 (2013). 10.1021/ar300314s 24245999

[36] G. G. Morbioli, N. C. Speller, and A. M. Stockton, “A practical guide to rapid-prototyping of PDMS-based microfluidic devices: A tutorial,” Anal. Chim. Acta 1135, 150–174 (2020). 10.1016/j.aca.2020.09.013 33070852

[37] J. Friend and L. Yeo, “Fabrication of microfluidic devices using polydimethylsiloxane,” Biomicrofluidics 4, 026502 (2010). 10.1063/1.3259624 20697575 PMC2917889

[38] T. Gervais, J. El-Ali, A. Günther, and K. F. Jensen, “Flow-induced deformation of shallow microfluidic channels,” Lab Chip 6, 500 (2006). 10.1039/b513524a 16572212

[39] B. S. Hardy, K. Uechi, J. Zhen, and H. Pirouz Kavehpour, “The deformation of flexible PDMS microchannels under a pressure driven flow,” Lab Chip 9, 935–938 (2009). 10.1039/B813061B 19294304

[40] D. W. Inglis, “A method for reducing pressure-induced deformation in silicone microfluidics,” Biomicrofluidics 4, 026504 (2010). 10.1063/1.3431715 20697573 PMC2917869

[41] K. Raj M and S. Chakraborty, “PDMS microfluidics: A mini review,” J. Appl. Polym. Sci. 137, 1–14 (2020). 10.1002/app.48958

[42] S. M. Recktenwald, K. Graessel, Y. Rashidi, J. N. Steuer, T. John, S. Gekle, and C. Wagner, “Cell-free layer of red blood cells in a constricted microfluidic channel under steady and time-dependent flow conditions,” Phys. Rev. Fluids 8, 074202 (2023). 10.1103/PhysRevFluids.8.074202

[43] C. W. Macosko, *Rheology: Principles, Measurements, and Applications* (Wiley-VCH, 1994).

[44] N. Takeishi, H. Ito, M. Kaneko, and S. Wada, “Deformation of a red blood cell in a narrow rectangular microchannel,” Micromachines 10, 199 (2019). 10.3390/mi10030199 30901883 PMC6470855

[45] S. Hénon, G. Lenormand, A. Richert, and F. Gallet, “A new determination of the shear modulus of the human erythrocyte membrane using optical tweezers,” Biophys. J. 76, 1145–1151 (1999). 10.1016/S0006-3495(99)77279-6 9916046 PMC1300064

[46] K. Sinha and M. D. Graham, “Dynamics of a single red blood cell in simple shear flow,” Phys. Rev. E 92, 042710 (2015). 10.1103/PhysRevE.92.042710 26565275

[47] T. Secomb, “Flow-dependent rheological properties of blood in capillaries,” Microvasc. Res. 34, 46–58 (1987). 10.1016/0026-2862(87)90078-1 3657604

[48] K. Tsukada, E. Sekizuka, C. Oshio, and H. Minamitani, “Direct measurement of erythrocyte deformability in diabetes mellitus with a transparent microchannel capillary model and high-speed video camera system,” Microvasc. Res. 61, 231–239 (2001). 10.1006/mvre.2001.2307 11336534

[49] G. Tomaiuolo and S. Guido, “Start-up shape dynamics of red blood cells in microcapillary flow,” Microvasc. Res. 82, 35–41 (2011). 10.1016/j.mvr.2011.03.004 21397612

[50] G. Tomaiuolo, M. Barra, V. Preziosi, A. Cassinese, B. Rotoli, and S. Guido, “Microfluidics analysis of red blood cell membrane viscoelasticity,” Lab Chip 11, 449–454 (2011). 10.1039/C0LC00348D 21076756

[51] V. Clavería, O. Aouane, M. Thiébaud, M. Abkarian, G. Coupier, C. Misbah, T. John, and C. Wagner, “Clusters of red blood cells in microcapillary flow: Hydrodynamic versus macromolecule induced interaction,” Soft Matter 12, 8235–8245 (2016). 10.1039/C6SM01165A 27714335

[52] A. Viallat and M. Abkarian, “Red blood cell: From its mechanics to its motion in shear flow,” Int. J. Lab. Hematol. 36, 237–243 (2014). 10.1111/ijlh.12233 24750669

[53] J. Mauer, S. Mendez, L. Lanotte, F. Nicoud, M. Abkarian, G. Gompper, and D. A. Fedosov, “Flow-induced transitions of red blood cell shapes under shear,” Phys. Rev. Lett. 121, 118103 (2018). 10.1103/PhysRevLett.121.118103 30265089

